# Zwischen Mimesis und Typus. Die rassenanthropologische Lektüre altägyptischer Menschendarstellungen im 19. und frühen 20. Jahrhundert†

**DOI:** 10.1002/bewi.201900021

**Published:** 2020-03-16

**Authors:** Felix Wiedemann

**Affiliations:** ^1^ Freie Universität Berlin Fachbereich Geschichts- und Kulturwissenschaften Fabeckstr. 23–25 Berlin 14195, Germany

**Keywords:** Geschichte der Archäologie, Ägyptologie, Kunstgeschichte und Rassenanthropologie, Orientalismus

## Abstract

Im Zuge der archäologischen Erschließung Ägyptens und des Vorderen Orients im 19. Jahrhundert kamen zahlreiche Menschendarstellungen aus den Kulturen des Altertums zum Vorschein, die auch jenseits der Altertums‐ und Kunstwissenschaften große Faszination auszuüben vermochten. Dabei wurden insbesondere altägyptische Statuen und Reliefs weniger als ästhetische Repräsentationen, sondern als mimetisch‐typologische Darstellungen wahrgenommen, die nicht nur bestimmte Individuen, sondern ganze Völker mit ihren charakteristischen physischen Merkmalen abbilden. Vor diesem Hintergrund avancierten Abbildungen dieser Bildwerke zu wichtigen visuellen Referenzen anthropologischer Publikationen und sollten die Konstanz vermeintlicher Rassenmerkmale und damit die Gültigkeit wissenschaftlicher Klassifikationen belegen. Anhand vornehmlich deutschsprachiger Publikationen aus dem 19. und frühen 20. Jahrhundert rekonstruiert der Beitrag die epistemologischen Voraussetzungen und bildästhetischen Prämissen der anthropologischen Lektüre altägyptischer Menschendarstellungen und geht der Frage nach, warum sich dieses Verfahren weitgehend auf Objekte aus den Kulturen des Alten Orients konzentrierte. Im Fokus steht hier die klassizistische Kunst‐ und Körperauffassung, die auf der einen Seite einer anthropologischen Lektüre von Objekten aus der klassischen Antike im Wege stand, auf der anderen aber dazu beitrug, den ägyptischen jenen mimetischen und typologischen Charakter zuzuweisen, der sie als visuelle Referenzen auch für die physische Anthropologie attraktiv machte.

Im Oktober 1817 machte Giovanni Belzoni (1778–1823), eine der schillerndsten Figuren der Archäologiegeschichte, seine wohl bedeutendste Entdeckung.^1^ Im ägyptischen Tal der Könige stieß er auf das Grab Pharao Setos I. (ca. 1323–1279 v. Chr.) – die längste, tiefste und vor allem die bestdekorierte Grabanlage der berühmten Nekropole. Der im britischen Auftrag agierende italienische Ausgräber war fasziniert von der intensiven Farbgebung und dem hervorragenden Zustand der ca. 3100 Jahre alten Wandgemälde und ließ sie durch seinen Zeichner Alessandro Ricci penibel nachzeichnen.^2^ Die bunten Darstellungen nutzte Belzoni schließlich nicht nur zur Illustration seines genrebildenden Reise‐ und Grabungsberichtes, sondern auch zur Ausschmückung eines spektakulären Nachbaus der Grabanlage, der 1821 in der *Egyptian Hall* am Londoner Piccadilly gezeigt wurde.^3^

**Figure 1:**
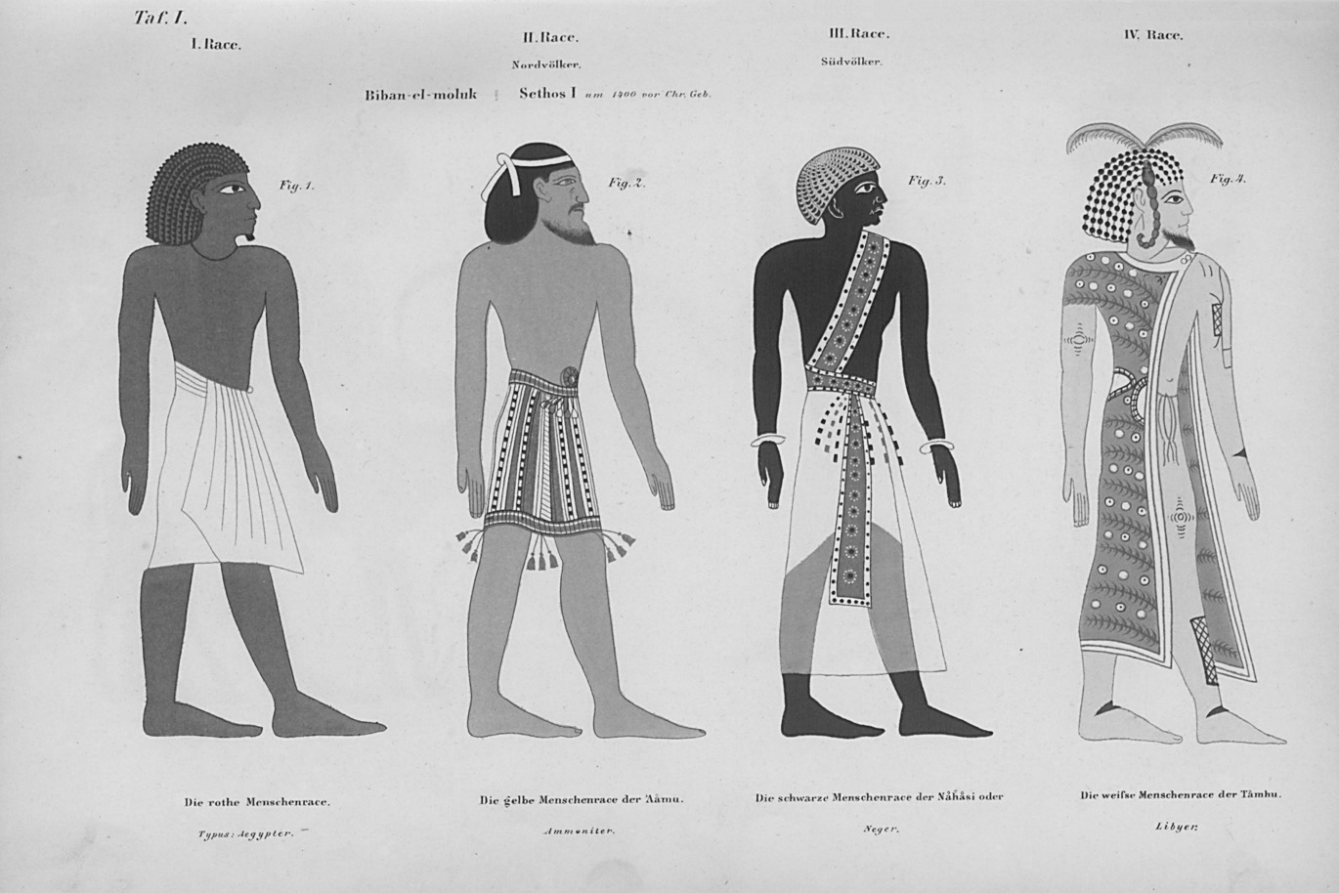
Die “vier Rassen” der Ägypter. Zusammenstellung aus dem Grabmal Sethos I. Hier aus einer Publikation des Ägyptologen Heinrich Brugsch von 1858 (Tafel 1).

Auf wessen letzte Ruhestätte er gestoßen war, wusste Belzoni allerdings nicht: Eine irreführende Übersetzung der Hieroglypheninschriften hatte ihn zur Überzeugung kommen lassen, es mit dem Grabmal des im Alten Testament erwähnten Pharaos Nechos II. aus der Mitte des ersten vorchristlichen Jahrtausends zu tun zu haben, der den judäischen König Josia bei Megiddo getötet haben soll (2 Kön 23, 29). Vor dem Hintergrund der biblischen Erzählung versuchte sich der Ausgräber selbst an einer Deutung und interpretierte die Figuren als Repräsentationen verschiedener Völker^4^: Die roten Figuren hielt er für ägyptische Aufseher über kriegsgefangene Äthiopier (schwarze Figuren), Perser (weiße Figuren) und Juden bzw. Judäer (gelbe Figuren).^5^ Als Identifizierungsmerkmal fungierte also jeweils die auffällige Farbgebung der Figuren, die wiederum die vermeintlich unterschiedliche Hautfarbe der genannten Völker anzeigen sollte. Mit anderen Worten, Belzoni übertrug die Hautfarbenklassifikation der modernen physischen Anthropologie bzw. Rassenanthropologie^6^ auf ein altägyptisches Bildwerk.

Zusätzliche Bestätigung erfuhr diese Deutung wenig später durch Jean‐François Champollion (1790–1832), Entzifferer der Hieroglyphen und Gründungsfigur der europäischen Ägyptologie. Im Unterschied zu Belzoni wusste Champollion die Grabanlage richtig zu datieren und hielt die Figuren auch nicht für Darstellungen konkreter Völker. Vielmehr glaubte er, es mit der Repräsentation einer fundamentalen Differenzierung der Menschheit nach charakteristischen physiologischen Merkmalen (Hautfarbe, Schädel, Nasen) zu tun zu haben: „Nous avons donc ici sous les yeux l'image des diverses *races d'hommes* connues des Égyptiens“.^7^ Dieser Ansicht schlossen sich nahezu sämtliche Ägyptologen des 19. Jahrhunderts an, so dass bald nur noch von den „vier Rassen“ die Rede war, nach denen die Ägypter die Menschheit unterteilt hätten.^8^ Die Ausstrahlungskraft des Bildes machte aber keineswegs an den Grenzen der Ägyptologie oder der Altertumswissenschaften halt. Vielmehr hielten die „vier Rassen“ auch umgehend Einzug in natur‐ und biowissenschaftliche Publikationen, schienen sie doch nicht nur die „Verschiedenheit der Rassen, sondern auch ihre Constanz“ und damit die Evidenz anthropologischer Klassifikationen eindrücklich zu untermauern.^9^ Der nationalsozialistische Anthropologe Egon Freiherr von Eickstedt (1892–1965) erkor die Ägypter sogar zu den Gründervätern der Rassenkunde und rühmte den „ausgeprägt wissenschaftlichen Geist der Altägypter“, mit dem diese die Bedeutung rassischer Differenz erfasst hätten. Entsprechend zieren die „vier Rassen“ die erste Seite seines voluminösen Hauptwerkes *Rassenkunde und Rassengeschichte der Menschheit* (1934).^10^

Die Interpretation der bunten Figuren aus dem Grab Setos I. als Repräsentation einer antiken Rassentypologie stellte lediglich den Auftakt einer umfassenden anthropologischen Auswertung altägyptischer Menschendarstellungen im 19. und frühen 20. Jahrhundert dar. Die Anthropologen, Archäologen, Ägyptologen, Bibelwissenschaftler, Historiker und Kunstgeschichtler der folgenden Generationen interessierten sich vor allem für die sogenannten Fremdvölkerdarstellungen auf den Reliefs der großen Tempelanlagen von Karnak und Medinet Habu, auf denen sich die Herrscher des Neuen Reiches ihrer tatsächlichen und vermeintlichen Siege über ihre Feinde rühmten.^11^ Wie bei Belzoni bedurfte die anthropologische Identifizierung aber immer der narrativen Kontextualisierung. So versuchte man an den Reliefs vor allem, aus der Bibel und der antiken Historiographie bekannte Völker wie die Hethiter oder Amoriter zu identifizieren und anthropologisch zu klassifizieren.^12^

Die Crux der anthropologischen Lektüre antiker Menschendarstellungen bestand darin, diese weder primär als ästhetische Objekte zu behandeln, die entsprechend mit ästhetischen Methoden und Begriffen zu untersuchen wären, noch als Porträts individueller Personen. Vielmehr galten sie als Ausdruck einer „Gruppencharakteristik“^13^, d. h. als typisierende Repräsentationen kollektiver Entitäten – sogenannter Völker und Rassen – mit ihren charakteristischen physischen Merkmalen. Die Attraktivität der Objekte basierte also auf der doppelten Prämisse ihres ebenso mimetischen wie typologischen Charakters. Konsequenterweise setzte man sie mit modernen anthropologischen Typenfotographien in Beziehung und wies ihnen eine quasi fotographische Evidenz zu. So zierten Abbildungen ägyptischer und altorientalischer Menschendarstellungen bald auch die Fotocollagen anthropologischer und rassenkundlicher Publikationen.^14^ Ein auffälliges Merkmal stellt jedoch das weitgehende Fehlen von entsprechenden Objekten aus der griechischen und römischen Antike in diesem Zusammenhang dar. Dass man diese offenkundig einer anthropologischen Lektüre für unzugänglich hielt, hatte auch Eickstedt unmittelbar im Anschluss an seine Wertschätzung der ägyptischen Rassendarstellungen herausgestellt: „Das klassische Altertum bedeutet dagegen einen Rückschritt.“^15^

Der Einsatz und die epistemische Funktion von Bildern, Zeichnungen und – vor allem – Fotographien in den Wissenschaften des 19. und frühen 20. Jahrhunderts stehen seit einigen Jahren im Fokus eines interdisziplinären Forschungsfeldes.^16^ Die anthropologische Lektüre von Abbildungen antiker Menschendarstellungen hat bisher aber weder im Rahmen der Geschichte der Anthropologie und Ethnologie,^17^ noch der Altertums‐ und Kunstwissenschaften genuine Aufmerksamkeit erfahren.^18^ Dabei handelte es sich keineswegs um eine bloße Skurrilität oder eine vor‐, pseudo‐ oder außerwissenschaftliche Spinnerei. Vielmehr muss von einem über die Fächergrenzen hinweg anerkannten Verfahren gesprochen werden, welches überdies einmal mehr vom trans‐ oder interdisziplinären Charakter wissenschaftlicher Rassendiskurse zeugt.^19^ Offenkundig besaß die Methode ein hohes Maß an Evidenz oder Einsichtigkeit: Kein Altertums‐, Kunst‐ oder Naturwissenschaftler musste jedenfalls begründen, wenn er^20^ unmittelbar von Statuen oder Reliefs auf das physische Erscheinungsbild vergangener Völker schloss. Warum aber, so fragt sich, vermochte ein solches Verfahren fächerübergreifend Evidenz zu entfalten? Und warum fokussierte sich das anthropologische Interesse nahezu ausschließlich auf Menschendarstellungen aus Ägypten und dem Vorderen Orient, während die in der zeitgenössischen Ästhetik so hochgeschätzten Statuen aus der klassischen Antike weitgehend unbeachtet blieben?

Wie ich im Folgenden zeigen möchte, lassen sich dieses Verfahren und die Grundannahmen, auf denen es basierte, nur verstehen, wenn man zentrale bildästhetische und kunsthistorische Prämissen und Narrative in Rechnung stellt, die ihren Hintergrund in der klassizistischen Kunstauffassung haben. Denn, so die hier verfolgte These, ebenso wie diese auf der einen Seite einer anthropologischen Lektüre von Objekten aus der griechischen Antike im Wege stand, so trug sie auf der anderen Seite dazu bei, den ägyptischen jenen mimetischen und typologischen Charakter zuzuweisen, der eine solche Lektüre erlaubte. In diesem Sinne gilt es zunächst, die maßgeblich durch Johann Joachim Winckelmann (1717–1768) geprägte Vorstellung einer nicht‐mimetischen oder „idealischen“ griechischen Kunst (1) und ihre Kontrastierung mit einer vermeintlich stärker abbildhaften ägyptischen Kunst zu skizzieren, die in den Kunst‐ und Altertumswissenschaften des 19. Jahrhunderts um die These einer typisierenden und quasi wissenschaftlichen Erfassung der Dinge durch die ägyptischen Künstler erweitert wurde (2). Vor diesem Hintergrund wird noch einmal auf die auch nach Einführung der Fotographie fortbestehende Bedeutung der ägyptischen Typendarstellung in der Rassenanthropologie zurückzukommen sein (3), bevor abschließend der um 1900 einsetzende Bruch mit den ästhetischen Prämissen skizziert wird, die der anthropologischen Lektüre antiker Menschendarstellungen zu Grunde lagen (4).

Vor dem Hintergrund der fächerübergreifenden Verbreitung des Verfahrens beziehe ich mich auf exemplarisch ausgewählte Publikationen von Altertumswissenschaftlern und Kunsthistorikern auf der einen, Naturwissenschaftlern und Anthropologen auf der anderen Seite aus dem Zeitraum vom späten 18. bis ins frühe 20. Jahrhundert, ohne freilich auch nur annähernd Vollständigkeit beanspruchen zu wollen. Wenn hier vor allem deutschsprachige Texte herangezogen wurden, so soll damit zudem keineswegs behauptet werden, die anthropologische Lektüre antiker Menschendarstellungen sei vornehmlich eine deutsche Angelegenheit gewesen. Abgesehen von pragmatischen Gründen rechtfertigt sich diese Beschränkung jedoch durch die zentrale Bedeutung deutschsprachiger Autoren bei der Formulierung jener kunsthistorischen und bildästhetischen Prämissen, auf denen die anthropologische Lektüre der Objekte beruhte – und genau um diese soll es im Folgenden gehen.

## 1. Die nicht‐mimetische Kunst der Griechen

Auf die normative Funktion der klassizistischen Kunst‐ und Körperästhetik bei der Differenzierung und Hierarchisierung menschlicher Varietäten oder Rassen seit dem 18. Jahrhundert ist vielfach hingewiesen worden.^21^ Zeichnungen (später auch Fotographien) griechischer und römischer Statuen fungierten in diesem Zusammenhang aber vornehmlich als visuelle Repräsentationen eines bestimmten Körper‐ und Rassenideals – und weniger als unmittelbare Porträts der antiken Völker, von denen man sich Aufschluss über deren tatsächliches physisches Erscheinungsbild versprochen hätte. Wenn etwa der als Gründungsfigur der modernen Rassenanthropologie geltende Johann Friedrich Blumenbach (1752–1840) als Referenzobjekt der sogenannten kaukasischen (europäischen oder weißen) Rasse auf eine für antik gehaltene Marmorbüste der Klythia aus dem Britischen Museum verwies,^22^ so wollte er damit vor allem unterstreichen, dass diese Varietät die „nach den europäischen Begriffen von Schönheit […] best gebildeten Menschen“ umfasse.^23^ In seiner ästhetischen Präferenz für die Kaukasier war Blumenbach stark durch die klassizistische Kunst‐ und Körperästhetik geprägt und mit den einschlägigen Schriften Winckelmanns eingehend vertraut.^24^ Bereits dieser aber hatte einer unmittelbaren anthropologischen Lektüre griechischer Statuen eine klare Absage erteilt und stets betont, dass es sich bei den griechischen Menschendarstellungen keineswegs um mimetische Abbildungen handele – auch nicht der Griechen selbst.

Winckelmann war ein aufmerksamer Rezipient der ethnographischen und anthropologischen Literatur seiner Zeit und unterzog vor allem den dem Menschen gewidmeten Band der *Histoire naturelle, générale et particulière* (1749) des französischen Naturforschers Georges‐Louis Leclerc de Buffon (1707–1788) einer intensiven Lektüre.^25^ Wie Buffon interessierte ihn dabei weniger die typologische Einteilung der Menschheit in Rassen als die Verteilung der Schönheit unter den Völkern. Im Rückgriff auf die Buffon'sche Klimatheorie zeigte er sich schließlich von der körperlichen Überlegenheit und „vorzügliche[n] Schönheit“ der – alten wie rezenten – Griechen überzeugt.^26^ Das bedeutete seiner Ansicht nach aber keineswegs, die griechischen Bildhauer hätten einfach nur die Menschen ihrer Umgebung abgebildet. Vielmehr, so Winckelmann, suchten sie „das Schöne aus [den] vielen schönen Körpern zu vereinigen“, die sie umgaben. Auf diese Weise vermochten sie es, von den ästhetischen Mängeln der natürlichen Objekte zu abstrahieren und prometheisch etwas zu schaffen, was schöner ist als die Natur selbst.^27^ Schönheit stellte in der klassizistischen Kunstauffassung also etwas Kompositorisches dar, das erst im menschlichen Geist Gestalt annimmt. So wollte Winckelmann die griechischen Götter‐ und Heldenstatuen auch keinesfalls mit einer bloß abbildenden Porträtkunst verglichen wissen:

Die Nachahmung des Schönen der Natur ist entweder auf einen einzelnen Vorwurf gerichtet, oder sie sammlet die Bemerkungen aus verschiedenen einzelnen, und bringet sie in eins. Jenes heißt eine ähnliche Copie, ein Portrait machen […]. Dieses aber ist der Weg zum allgemeinen Schönen und zu idealischen Bildern desselben; und derselbe ist es, den die Griechen genommen haben.^28^

Demgemäß lassen sich bei Winckelmann zwei verschiedene Arten der Naturnachahmung unterscheiden: eine mimetisch angelegte Variante, die auf eine exakte Kopie einzelner Objekte mit all ihren Makeln abzielt, und eine idealische Variante, die sich vom empirischen Einzelkörper löst und die Schönheit selbst darzustellen imstande ist. Als Objekte einer auf das tatsächliche – mitunter auch hässliche – physische Erscheinungsbild abzielenden anthropologischen Lektüre kamen nach den Prinzipien idealischer Schönheit gestaltete Kunstwerke daher nicht in Frage.

Zwar konnte die klassizistische Ästhetik im 19. Jahrhundert keine uneingeschränkte Geltung mehr beanspruchen,^29^ und die sich im Anschluss an (und in Abgrenzung von) Hegel etablierende Kunstgeschichtsschreibung löste sich zunehmend von der Fixierung auf die griechische Kunst.^30^ Dass aber, wie es auch bei Hegel heißt, wahre Kunst etwas „aus dem Geiste geborene[s]“ darstelle, dessen Zweck „in etwas anderem als in der bloß formellen Nachahmung des Vorhandenen“ liegen müsse, sich also nicht in bloßer Mimesis erschöpfe, blieb eine konstitutive Vorstellung der nachfolgenden Generationen.^31^

## 2. Der ägyptische Naturalismus

Folgt man hingegen den einschlägigen kunsthistorischen Werken des 19. Jahrhunderts, verhielt es sich bei der altägyptischen Kunst genau anders herum: Hier schien das bloß Mimetische zu dominieren, während das entscheidende Moment wahrer Kunst – die Überschreitung der Natur auf ein Ideal hin – den Ägyptern ebenso wie anderen Völkern des Alten Orients abgesprochen wurde. Bei allem Lob für die „Vollendung der technischen Ausführung“ vermisste nicht nur Hegel an den ägyptischen Statuen das „Wirken und Weben des Geistes“.^32^ In Franz Kuglers (1808–1858) *Handbuch der Kunstgeschichte* (1842), einem der meist gelesenen kunsthistorischen Werke des 19. Jahrhunderts, heißt es entsprechend:

Die körperliche Form ist den Aegyptern eben nichts als körperliche Form; davon, dass sie zugleich an sich der Ausdruck des Geistes sei, dass sie wesentlich nur dazu diene, den Geist zur Erscheinung zu bringen, wissen sie nichts.^33^

Hier stand freilich nicht nur Hegel Pate. In Abgrenzung zur Ägyptenschwärmerei des 17. und frühen 18. Jahrhunderts^34^ hatte bereits Winckelmann die ägyptische Kunst entschieden abgewertet und argumentiert, die Ägypter hätten bloß „die Natur nachgeahmt […], wie sie dieselbe fanden“,^35^ ohne dabei – wie die Griechen – etwas zu erschaffen, „das nur irgend einen Begriff vom Schönen geben könnte“.^36^ So war es nur konsequent, dass seine Abwertung der ägyptischen Kunst mit einer Abwertung auch der ägyptischen Körper einherging. Die ägyptischen Gesichter etwa erschienen ihm deshalb unansehnlich, weil sie vom „griechischen Profil“^37^ abwichen: „Die Augen sind gegen die Nase abwärts geneigt, die Wangen aufgerieben, der Mund ist aufgeworfen und das Kinn kurz“.^38^ Als steinerne Zeugnisse für die Hässlichkeit der Ägypter fungierten allein die ihm bekannten Menschendarstellungen. Denn im Unterschied zu den idealen griechischen Statuen schien der mimetische Charakter dieser Monumente solche Rückschlüsse auf das Reale nunmehr zu erlauben. Es war also der Kunstschriftsteller Winckelmann – und nicht etwa ein Vertreter der frühen Rassenanthropologie –, der die ägyptischen Menschendarstellungen als erster einer anthropologischen Lektüre unterzog.

Im Grunde aber hatten weder Winckelmann noch Hegel eine wirkliche Vorstellung davon, wovon sie schrieben, denn die meisten Objekte aus dem alten Ägypten kamen erst nach und nach im Zuge der archäologischen Erschließung des Nillandes seit dem frühen 19. Jahrhundert ans Licht.^39^ Der Topos der mimetischen Kunst blieb davon aber zunächst unberührt. Denn auch wenn den ägyptischen und altorientalischen Objekten nunmehr ein größerer – und bisweilen auch positiver – Stellenwert eingeräumt wurde, hielten sich zentrale Figuren und Narrative der klassizistischen Kunsttheorie bis in das späte 19. Jahrhundert ungebrochen. Der vermeintliche Abbildcharakter ägyptischer Bilder wurde nunmehr auch als „Naturalismus“ oder „Realismus“ bezeichnet und auf diese Weise mit den entsprechenden Strömungen der Gegenwartskunst in Beziehung gesetzt.^40^ Entsprechend reflektierten die Bewertungen ägyptischer Statuen und Reliefs immer auch aktuelle kunst‐ und kulturpolitische Debatten. Während vor allem unter den Historikern und klassischen Archäologen ein auf die klassische Antike fokussierter kunst‐ und kulturpolitischer Konservatismus vorherrschte,^41^ zeigten sich Kunsthistoriker und Orientwissenschaftler offener und wussten den „frischen“^42^ oder gar „kongenialen Realismus“^43^ der ägyptischen Plastiken und Reliefs durchaus zu schätzen (ohne dabei die Überlegenheit der griechischen Kunst in Frage zu stellen). Was die Kunst betrifft, so schienen Ägypter und Griechen einfach unterschiedlichen Prinzipien oder Idealen zu folgen, wie etwa der Ägyptologe Wilhelm Spiegelberg (1870–1930) prägnant herausstellte:

Für den ägyptischen Künstler war das Höchste die möglichst genaue Wiedergabe seines Vorwurfs […]. Die Schönheit, das Ideal der Griechen, hat in Ägypten stets im Schatten der Wahrheit gestanden, und der Realismus entspricht dem ägyptischen Genius wie den religiösen Anschauungen des Volkes.^44^

Wenn in Bezug auf ägyptische Menschendarstellungen von „frische[r] Naturwahrheit“^45^ oder „packender Lebenswahrheit“^46^ die Rede war, so bezog sich das in der Regel weniger auf die vermeintlich abbildhaften Porträtdarstellungen einzelner Personen, wie man sie etwa an den berühmten Statuen des Rahotep und der Nofret aus der Mitte des dritten vorchristlichen Jahrtausends bewunderte.^47^ Seit dem späten 19. Jahrhundert unterschieden die Ägyptologen und Kunsthistoriker vielmehr zwischen einem formalisierten, repräsentativen und autoritativen „Hofstil“ und einem freieren „Volksstil“, der vor allem bei der Darstellung typischer Alltagsszenen verwendet worden sei.^48^ Genau aus diesem Grund genoss die sogenannte Volkskunst auch die größere Wertschätzung: Denn was man hier zum Ausdruck kommen sah, war ein über das bloße Abbilden von Einzelheiten hinausgehendes Erfassen des Typischen oder Charakteristischen, das im Wesentlichen dem von Lorraine Daston und Peter Galison beschriebenen wissenschaftlichen Bildideal der „Naturwahrheit“ entsprach.^49^ Die meisten Kunsthistoriker und Ägyptologen lobten denn auch ausdrücklich die Fähigkeit der ägyptischen Künstler, „mit wenigen Strichen charakteristische Abbilder zu schaffen, überall das Wesentliche zu sehen“^50^ und auf diese Weise „die charakteristischen Momente der dargestellten Scenen zur möglichst klaren Anschauung zu bringen.“^51^ Wie die idealische Darstellung abstrahiert zwar auch die typisierende Darstellung von individuellen Merkmalen, die Ausrichtung ist jedoch eine gänzlich andere: Stellt jene eine ästhetisierende Überschreitung des Realen dar, zielt diese darauf ab, das Reale adäquater und genauer zu erfassen, als es eine rein mimetische Darstellung vermag.

Aus dieser Perspektive erschien auch die bereits auf Platon zurückgehende Vorstellung von der Unwandelbarkeit der ägyptischen Kunst und ihrer, wie Hegel prägnant festhielt, „statarische[n] Treue“ gegenüber überlieferten Konventionen in neuem Licht.^52^ Ein schematisierender Blick, der vom Individuellen abstrahiert, gehört schließlich ebenso zu den unabdingbaren Vermögen bei der Erfassung des Typischen wie eine schematisierte Darstellungsweise die Voraussetzung des typologischen Vergleichs darstellt.^53^ Je strikter die Regeln bei der Gestaltung der Objekte (Perspektive, Körperproportionen, Haltung, Profil etc.) ausfallen, desto besser lassen sie sich in Beziehung zueinander setzen. Für typologische Vergleiche taugen die von Hegel konstatierte „Unbeweglichkeit“ und der „leblose Ernst“ der ägyptischen Skulpturen mithin weitaus mehr als die vermeintliche „Freiheit und Lebendigkeit“ der griechischen Skulpturen.^54^ Wollte man etwa die typischen Charakterzüge der von den Ägyptern dargestellten Personen und Gruppen erfassen, so stellte die von der klassizistischen Ästhetik beanstandete „Gleichförmigkeit und Gesetzlichkeit in der Körperbildung“^55^ der Statuen ebenso einen Vorteil dar wie ihre „Frontalität“, die der dänische Kunsthistoriker Julius Henrik Lange (1838–1896) zunächst als Ausdruck einer – im Vergleich zur griechischen Kunst – primitiveren kunsthistorischen Stufe beschrieben (und bemängelt) hatte.^56^ Für die Erstellung von Vergleichstypologien aber schien die Frontalansicht hervorragend geeignet, ähnelte sie doch nicht von ungefähr der anthropometrischen Fotographie, wie sie sich in der Kriminologie und physischen Anthropologie am Ende des 19. Jahrhunderts etablierte.^57^ Vor diesem Hintergrund mehrten sich nunmehr auch in der kunsthistorischen Literatur Hinweise auf die vermeintlich vortreffliche Erfassung rassischer Merkmale auf den ägyptischen Reliefs: „[M]it scharfem Blick werden die Rasseneigenschaften der verschiedenen Völkerstämme, der Nubier, Libyer, Beduinen, Semiten u. a. […] wiedergegeben.“^58^

Anders als noch bei Winckelmann und Hegel erschien der ägyptische Bildhauer im späteren 19. Jahrhundert also nicht mehr nur als bloßer Handwerker.^59^ Wenn – der dominierenden kunsthistorischen Auffassung des 19. Jahrhunderts zufolge – das Streben nach dem Ideal den griechischen Bildhauer zum Künstler machte, so war sein ägyptischer Vorläufer mit seiner mimetisch‐typisierenden Darstellungsweise im Grunde ein Wissenschaftler. Der nunmehr zu einem festen Attribut der Ägypter avancierende „nüchterne Verstand“^60^ – im Zeitalter des wissenschaftlichen Positivismus eine zweifellos geschätzte Eigenschaft – korrespondierte dabei mit der zunehmenden Entzauberung oder „Banalisierung“ des Pharaonenreiches im 19. Jahrhundert im Zuge seiner wissenschaftlichen Erschließung.^61^ Im Unterschied zu den „ägyptomanischen“ Deutungen früherer Epochen galten die altägyptischen Bildwerke nicht mehr als Träger einer „Symbolik der Erhabenheit“^62^ oder esoterischer Geheimlehren, sondern als Ausdruck präziser Naturbeobachtung. Auf diese Weise aber schienen gleichsam auch ihre ästhetischen Defizite erklärlich, so dass die ägyptische Kunst in vielen kunst‐ und altertumswissenschaftlichen Darstellungen des 19. Jahrhunderts einen paradoxen Status einnahm, wie der Hegel‐Schüler Carl Schnaase (1798–1875) betonte: „Die Vorzüge dieser Kunst hängen also mit ihren Mängeln zusammen.“^63^ Erst vor dem Hintergrund dieser ambivalenten Stellung der ägyptischen Werke – ihrer ästhetischen Geringschätzung bei gleichzeitiger Anerkennung ihres wissenschaftlichen Wertes als mimetisch‐typisierende Darstellungen – wird verständlich, warum die ägyptischen Menschendarstellungen auch für die Rassenanthropologie attraktiv wurden.

## 3. Antike Typendarstellung und anthropologische Fotographie

Von den frühen Rassenanthropologen hat sich vor allem Blumenbach wiederholt mit Objekten aus dem alten Ägypten – mit Mumien, aber auch mit Statuen und Reliefs – beschäftigt.^64^ Anders jedoch als die vermeintlich antike Klythia zog er die „altägyptischen Kunstwerke“ nicht als Repräsentationen idealer körperlicher Schönheit heran. Vielmehr glaubte er, durch präzises Studium der Objekte die „verschiedenen Nationalphysiognomien“ der alten Ägypter rekonstruieren zu können, sah in ihnen also mimetisch‐typologische Darstellungen ihres körperlichen Erscheinungsbildes.^65^

Im Zuge ihrer archäologischen Erschließung und musealen Präsentation setzte schließlich bald eine systematische anthropologische Auswertung der ägyptischen Menschendarstellungen ein. Bereits in den Publikationen europäischer und nordamerikanischer Rassenwissenschaftler aus der Mitte des 19. Jahrhunderts haben sie einen festen Platz. Besonders berüchtigt in dieser Hinsicht war das einflussreiche Werk *Types of Mankind* (1854), das der amerikanische Anthropologe Josiah Nott (1804–1873) zusammen mit dem britischen Ägyptologen George Gliddon (1809–1857) verfasst hatte. Der Rekurs auf altägyptische Menschendarstellungen sollte hier nicht nur die Unveränderbarkeit und Konstanz rassischer Merkmale, sondern vor allem den polygenetischen Ursprung des Menschen und damit die Nichtverwandtschaft von Schwarzen und Weißen belegen.^66^ Die Autoren versuchten zwar auch, einzelne Pharaonen anhand ihrer vermeintlichen Porträts rassenanthropologisch zu bestimmen,^67^ wie bei den meisten Rassenanthropologen – und den Kunsthistorikern – lag der Interessenfokus jedoch auf den typisierenden Darstellungen. Ihre unangefochtene Stellung als bildliche Zeugnisse rassischer Konstanz und „Höhepunkt ethnographischer Charakteristik“^68^ im Altertum behielten die ägyptischen Menschendarstellungen auch dann noch, als sie im Zuge der archäologischen Erschließung der altmesopotamischen Kulturen visuelle Konkurrenz erhielten.^69^ Zwar wurden auch Statuen und Reliefs aus den Kulturen des Zweistromlandes als mimetisch‐typologische Darstellungen von Volks‐ und Rassetypen gedeutet und entsprechenden Lektüren unterzogen. In der anthropologischen Literatur aber erlangten sie niemals die gleiche Prominenz wie die ägyptischen Skulpturen und Reliefs. Beklagt wurde hier vor allem eine mangelnde Vielfalt der dargestellten Völker und Typen: An einer panoramaartigen Typisierung der Völker und Rassen, wie man sie auf den ägyptischen Monumenten bewunderte, schienen weder die Sumerer, noch die Babylonier, Assyrer oder Hethiter interessiert gewesen zu sein.^70^

Unter den deutschen Anthropologen, die sich um 1900 intensiver mit den ägyptischen Menschendarstellungen beschäftigen, sticht vor allem Gustav Fritsch (1838–1927) hervor. Dies ist nicht zuletzt deswegen bemerkenswert, weil Fritsch zugleich einer der Pioniere und Theoretiker der anthropologischen Fotographie war.^71^ Als maßgeblich sollte sich in diesem Zusammenhang nicht nur seine grundlegende Unterscheidung zwischen „ethnologischen“ und „physiognomischen Aufnahmen“ erweisen, sondern vor allem die praktischen Instruktionen zur Erstellung standardisierter und damit vergleichbarer Bilder.^72^ Auf diese Weise versuchte er, auf ein zentrales Problem der anthropologischen Fotographie eine Antwort zu geben. Genau wie die vermeintlich mimetische Porträtkunst vermag nämlich die „mechanische Objektivität“^73^ der Fotographie, das Typische oder Charakteristische nur unzureichend festzuhalten: Auf der Fotografie eines individuellen Gegenstandes oder eines Menschen erscheinen zunächst alle Elemente gleich wichtig; Typen werden jedoch aufgrund *ausgewählter* Merkmale konstituiert, die auf Fotographien mitunter kaum erkennbar sind. Bei der Hervorhebung jener Details, die den Typus repräsentieren, sind naturgetreue Zeichnungen – oder eben auch antike Statuen und Reliefs mimetisch‐typologischen Charakters – den Fotographien also überlegen. Auch wenn bereits um 1900 verschiedene Verfahren entwickelt wurden, Völker‐ und Rassentypen auch fotographisch sichtbar zu machen, wie Amos Morris‐Reich in seiner Studie über die Rassenfotographie gezeigt hat, so vermochte die Fotographie die Zeichnung ebenso wenig gänzlich aus der Rassenkunde zu verdrängen wie die Abbildung antiker Menschendarstellungen.^74^

Gerade diese zirkulierten in den meisten Darstellungen um 1900 nicht mehr als Zeichnungen, sondern in fotographischer Form. Nunmehr wurden sogar archäologische Expeditionen mit dem Ziel ausgesandt, die ägyptischen Menschendarstellungen fotographisch zu erfassen. Ein entsprechendes Projekt führte 1886/87 William Matthew Flinders Petrie (1853–1942), eine der Gründungsfiguren der britischen Ägyptologie und prähistorischen Archäologie, an den Nil und stellte den Auftakt seiner lebenslangen Beschäftigung mit der Rassengeschichte des alten Ägyptens dar.^75^ In seiner viel rezipierten Publikation *Racial Photographs from the Egyptian Monuments* (1887) finden sich jedoch keine Aufnahmen der ägyptischen Bildwerke selbst: Vielmehr handelte es sich um Fotographien von Gipsabgüssen, die er von vermeintlich charakteristischen Kopfdarstellungen hatte anfertigen lassen. Die anthropologische Auswertung setzte also nicht nur die Herauslösung einzelner Elemente aus ihren bildlichen Kontexten voraus, sondern basierte auf einer fundamentalen medialen Transformation. Das wohl umfangreichste und am besten ausgestattete Unterfangen dieser Art, die von Eduard Meyer (1855–1930), dem wohl einflussreichsten deutschen Altertumswissenschaftler seiner Zeit, initiierte *Expedition nach Ägypten zur Erforschung der Darstellungen der Fremdvölker* 1912/13, zielte demgegenüber zwar auf die fotographische Erfassung ganzer Bildwerke.^76^ Vor dem Hintergrund der einsetzenden Kriegs‐ und Krisenperiode wurden diese Fotographien jedoch niemals zusammenhängend publiziert und hinterließen daher wenige Spuren in den zeitgenössischen anthropologischen Lektüren.

## 4. Ausblick

Beispiele für die anthropologische Lektüre altägyptischer und altorientalischer Menschendarstellungen lassen sich in wissenschaftlichen Publikationen bis in die 1980er Jahre finden.^77^ Noch Anfang der 1970er Jahre etwa interpretierte der deutsche Ethnologe Klaus E. Müller die eingangs diskutierte Darstellung aus dem Grabmal Setos I. als „entscheidende[n] Schritt auf dem Weg zu einer systematischen Rassengliederung der Menschheit“.^78^

Dennoch war die ästhetische und kunsthistorische Prämisse der Methode, nämlich die These vom mimetisch‐typologischen Charakter der Bildwerke, bereits um 1900 ins Wanken geraten. Auf der einen Seite geriet die klassizistische Kunstauffassung mit ihren fundierenden entwicklungstheoretischen Narrativen zunehmend unter Druck und drohte selbst ihre Deutungshoheit über die „idealischen“ Objekte aus der griechisch‐römischen Antike einzubüßen.^79^ Auf der anderen Seite aber begann sich vor allem die Wahrnehmung der ägyptischen Werke zu ändern.^80^ Anstatt ihren defizitären Status gegenüber griechischen Objekten herauszustellen, wies man ihnen nunmehr einen ästhetischen Eigenwert zu und suchte ihre Prinzipien und Gestaltungsregeln zu verstehen. Eine wichtige Rolle spielten hier etwa die unter dem Titel *Abstraktion und Einfühlung* (1907) erschienene Dissertation des Kunsthistorikers Wilhelm Worringer (1881–1965)^81^ und die Studie über *Die Plastik der Ägypter* (1914) der deutsch‐jüdischen Kunstwissenschaftlerin Hedwig Fechheimer (1871–1942).^82^ In den 1920er Jahren unterzog dann der Ägyptologe Heinrich Schäfer (1868–1957) die ägyptische Reliefkunst einer eingehenden Untersuchung und betonte gerade deren nicht‐mimetischen Charakter.^83^ Im Hintergrund dieser – jeweils ganz unterschiedliche Agenden verfolgenden – Neubewertungen der Objekte stand dabei nicht zuletzt die ästhetische Transformation der Moderne: Nicht nur Fechheimer, sondern viele jener modernen Künstler, die mit den naturalistischen Darstellungsprinzipien der europäischen Kunsttradition gebrochen hatten, wähnten sich in „geistige[r] Kontinuität mit Ägypten“^84^ oder ließen sich von der „Geradaufsichtigkeit“^85^ der ägyptischen Reliefs, wie sie Schäfer beschrieben hatte, inspirieren. Der ägyptische Bildhauer erschien nunmehr weder als bloßer Handwerker noch als verkappter Wissenschaftler, sondern als Künstler. In dem Moment aber, in dem die ägyptischen Bildwerke primär als ästhetische Objekte von eigenem Wert wahrgenommen wurden, verloren sie ihre Evidenz als mimetisch‐typologische Menschendarstellungen und kamen als Objekte anthropologischer Studien prinzipiell nicht mehr in Frage.^86^

## Footnotes

1 Zur Biographie Noël Hume 2011. Zum Kontext u. a. Jasanoff 2006, S. 247–256; McGeough 2015, S. 84–103.

2 Bedauerlicherweise sind die originalen Wandmalereien erheblich beschädigt worden, so dass das Grab 1978 für die Öffentlichkeit geschlossen werden musste. Siehe Hornung und Burton 1991.

3 Belzoni 1820b, S. VI–VIII; ferner Belzoni 1820a, S. 233–242. Zur Ausstellung Pearce 2000; Thomas 2012.

4 Der Lesbarkeit halber wird im Folgenden bei historisch belasteten Klassifikationsbegriffen wie *Volk* und *Rasse* auf eine Markierung verzichtet.

5 Belzoni 1820a, S. 242–243. Siehe auch die instruktive Skizze der Rezeption des Bildes bei Keevak 2011, S. 13–22.

6 Der Begriff *Anthropologie* wird im Folgenden im Sinne seiner vornehmlichen Bedeutung im deutschsprachigen Raum des 19. Jahrhunderts verwendet, d. h. als physische Anthropologie oder Rassenanthropologie, gleichwohl ältere und konkurrierende Bedeutungen (im Sinne einer philosophischen Anthropologie oder als Synonym für Völkerkunde) weiterhin kursierten. Zur Begriffsgeschichte Vermeulen 2015, S. 1–38. Zu den differenten Verwendungen des Begriffs in den europäischen Wissenschaften siehe die Beiträge in Barth 2005.

7 Champollion 1833, S. 248. Siehe hier auch die physiologischen Beschreibungen (S. 248–250). Zu Champollion gibt es eine umfangreiche Literatur. Siehe zuletzt die Biographie von Robinson 2014.

8 Wiedemann 1904, S. 64–65; ferner u. a. Champollion‐Figeac 1839, S. 57; Wilkinson und Birch 1878, S. 2; Budge 1906, S. 151; Wiedemann 1920, S. 27–28. Hier sind freilich die vielschichtigen Bedeutungen zu berücksichtigen, die der Rassenbegriff nicht nur in englisch‐ und französischsprachigen, sondern auch in deutschsprachigen Publikationen des 19. Jahrhunderts hatte: Weit davon entfernt, allein eine Kategorie der physischen Anthropologie darzustellen, waren die Grenzen zu anderen Kollektivbezeichnungen wie *Volk* und *Nation* (bzw. *peoples* und *nations*) niemals eindeutig. Anstatt den zeitgenössischen Wissenschaftlern aber eine prä‐ oder gar pseudowissenschaftliche Vermischung anthropologischer mit sprachlichen und ethnographischen Kategorien vorzuwerfen, scheint es sinnvoller, die verschiedenen Merkmalstypen als Indizien zu begreifen, die für die Existenz kollektiver Akteure (seien sie als Völker, Rassen oder Nationen bezeichnet) angeführt wurden und sich dabei wechselseitig bestätigten. Erhellend hierzu Hutton 2005, S. 94–98; ferner Wiedemann 2017. In diesem Sinne geht es im Folgenden um die Differenzierung kollektiver Entitäten nach (dargestellten) körperlichen Merkmalen – unabhängig davon, wie diese bezeichnet wurden und welche Indizien für ihre Existenz darüber hinaus in Anschlag gebracht wurden.

9 Vogt 1863, S. 227.

10 Eickstedt 1934, S. 1–3. Weitere Abbildungen u. a. bei Nott und Gliddon 1854, S. 85; Haddon 1909, S. 2; Seyffert 1913, S. 53; Winchell 1888, S. 199; Haddon 1910, S. 10.

11 Zu diesen Bildwerken u. a. Roth 2015.

12 Exemplarisch dazu Sayce 1893; Luschan 1922. Zu diesen Beispielen Wiedemann 2010.

13 Eickstedt 1934, S. 2.

14 Exemplarisch dazu Luschan 1922; Günther 1929.

15 Eickstedt 1934, S. 3.

16 Exemplarisch dazu die Beiträge in Geimer 2002; Wolf 2003; Wöhrer 2015; Hinterwaldner und Buschhaus 2006.

17 Zur anthropologischen Fotographie u. a. Edwards 2001; Theye 2004; Pinney 2011; Morris‐Reich 2016.

18 Zur archäologischen Fotographie Moser 1998; Bohrer 2011; Klamm 2017 sowie die Beiträge in Smiles und Moser 2005. In fachhistorischen Arbeiten zur Ägyptologie und Archäologie sind rassenhistorische Deutungen antiker Objekte zwar wiederholt problematisiert worden (siehe u. a. Trigger 2006, insbes. S. 166–210; Challis 2013; Riggs 2017; Voss 2016), ohne jedoch, dass die anthropologische Lektüre und ihre epistemologischen Voraussetzungen systematisch untersucht worden wären.

19 Siehe hierzu die Beiträge in Morris‐Reich und Rupnow 2017.

20 Die gennannten Wissenschaften wurden im fraglichen Zeitraum nahezu ausschließlich von Männern betrieben. Daher werden im Folgenden nur männliche Formen verwendet mit dem ausdrücklichen Hinweis, dass es sich nicht um das generische Maskulinum handelt.

21 Siehe u. a. Mosse 1996 [1978], S. 143–160; Volland 1992; Potts 1994, insbes. S. 159–163; Marchand 1996, S. 7–16; Sünderhauf 2004; Meijer 1999, S. 101–166; Bindman 2002.

22 Blumenbach 1790, S. 5. Hierzu grundlegend Klatt 2008. Zur Deutungsgeschichte des Objekts Lehmann 2016. Zur Kategorie der kaukasischen Rasse Baum 2006.

23 Blumenbach 1788, S. 61.

24 Siehe u. a. Blumenbach 1811, S. 129–130.

25 Buffon 2009 [1749]. Zur Buffon‐Rezeption im deutschsprachigen Raum Dougherty 1990. Zu Winckelmanns ethnographischen und anthropologischen Studien grundlegend Décultot 2004; Franke 2006.

26 Winckelmann 2016 [1755], S. 59. Zur Klimatheorie instruktiv Fink 1987.

27 Winckelmann 2002 [1764], S. 256.

28 Winckelmann 2016 [1755], S. 62.

29 Pointiert hierzu Sünderhauf 2004, S. 46–54.

30 Zur Bedeutung Hegels für die Kunstgeschichtsschreibung des 19. Jahrhunderts Gombrich 1977; Wyss 1989; Prange 2004, S. 71–94. Zur Geschichte der Kunstgeschichte allgemein Dilly 1979; Kauffmann 1993; Locher 2001.

31 Hegel 2017, S. 14, 69.

32 Hegel 2018, S. 448, 452. Zu Hegels Bild der ägyptischen Kunst Wyss 1989, S. 18–30.

33 Kugler 1842, S. 62–63. Zu Kugler siehe die Beiträge in Espagne et al. 2010.

34 Zur frühneuzeitlichen „Ägytomanie“ u. a. Curl 1994 sowie die Beiträge in Seipel 2000.

35 Winckelmann 2002 [1764], S. 56.

36 Winckelmann 2014 [1767], S. 76. Zu Winckelmanns Ägypten‐Bild Syndram 1990, S. 72–80; ferner die Beiträge in Kunze et al. 2003. Allerdings werden in diesem Band die problematischen Aspekte einfach ausgeblendet.

37 Winckelmann 2002 [1764], S. 326.

38 Winckelmann 2019 [1760], S. 54. Auch wenn es ihm nicht um Rasse, sondern um Schönheit ging, müssen Winckelmanns teilweise deutlich abwertenden Beschreibungen der Körper anderer Völker durchaus im Kontext des europäischen Rassismus gelesen werden. Hierzu Michaud 2017; ferner die in Anm. 22 angegebene Literatur.

39 Hierzu u. a. Jasanoff 2006, S. 211–306. Zur musealen Darstellung und Rezeption u. a. Moser 2006; Riggs 2017.

40 Terminologischer Vorreiter war hier vermutlich der Ägyptologe Adolf Erman (1854–1937). Siehe Erman 1885, S. 535. Für die Kunstgeschichte u. a. Springer 1898, insbes. S. 12–14; Lübke und Semrau 1908, S. 31–43.

41 Hierzu Sünderhauf 2004, S. 21–46.

42 Lübke und Semrau 1908, S. 43.

43 Spiegelberg 1903, S. 26.

44 Ebenda 1903, S. 29. Zum Autor Gertzen 2017, S. 166–169.

45 Springer 1881, S. 6.

46 Spiegelberg 1903, S. 20.

47 Zur Problematik des Porträtkonzepts in diesem Kontext Widmaier 2017, S. 322–373.

48 Spiegelberg 1903, S. 22–23. Es handelte sich also um eine Differenzierung anhand der Motive und weniger der sozialen Funktion der Kunst. Eingeführt worden war diese Unterscheidung vermutlich wiederum von Erman 1885, S. 535; ferner u. a. Springer 1898, S. 11–12; Lübke und Semrau 1908, S. 31–32.

49 Daston und Galison 2002, S. 35–57; dies. 2007, S. 59–119.

50 Spiegelberg 1903, S. 20.

51 Kugler 1842, S. 62–63.

52 Platon (Nomoi) 656d–656e; Hegel 2018, S. 448–449.

53 Erhellend in diesem Zusammenhang jetzt Meyer 2019, S. 11–38.

54 Hegel 2018, S. 450–452.

55 Schnaase 1843, S. 431.

56 Lange 1899, S. 7–31, insbes. S. XII.

57 Hierzu u. a. Regener 1999; Sekula 2003; Meyer 2019 sowie die in Anm. 18 angegebene Literatur.

58 Lübke und Semrau 1908, S. 34; Springer 1898, S. 27.

59 Einschlägig Winckelmann 2002 [1764], S. 62; Hegel 2018, S. 449.

60 Kugler 1842, S. 37; Spiegelberg 1903, S. 28; Lübke und Semrau 1908, S. 12.

61 Prägnant hierzu Marchand 2000.

62 Hegel 2017, S. 478.

63 Schnaase 1843, S. 444.

64 Blumenbach 1794; ders. 1811, S. 45–144.

65 Blumenbach 1811, S. 7, 129–130.

66 Nott und Gliddon 1854. Hierzu Kidd 2006, S. 121–167.

67 Nott und Gliddon 1854, S. 145–149.

68 Oberhummer 1904, S. 277.

69 Zur Grabungsgeschichte u. a. Larsen 2010; Fontan und Chevalier 1994; Mairs und Muratoc 2015. Zur Rezeption der altmesopotamischen Bildwerke Bohrer 2003; Malley 2012; McGeough 2015.

70 Siehe die Argumentation bei Oberhummer 1904, S. 278.

71 Fritsch 1904b; ders. 1904c; ders. 1904a.

72 Fritsch 1875, S. 607–613. Hierzu grundlegend Theye 2015.

73 Siehe dazu das gesamte Kapitel bei Daston und Galison 2007, S. 121–200.

74 Morris‐Reich 2016.

75 Petrie 1887. Hierzu Challis 2013, S. 85–106; Drower 1985, S. 127–152.

76 Meyer 1913. Zu Meyers Rassenbegriff v. a. Hauser 2005.

77 Exemplarisch Nagel 1987.

78 Müller 1972, S. 25.

79 Hierzu grundlegend Marchand 1996; Sünderhauf 2004.

80 Zu diesem Bruch siehe die instruktive Skizze bei Marchand 2000; ausführlich Schreiber 2018, S. 59–110.

81 Worringer 2004 [1907]. Die Literatur zu Worringer ist kaum zu überschauen. Siehe zuletzt die Beiträge in Gramaccini und Rössler 2012. Erhellend zu Worringers Ägypten‐Bild Schreiber 2018, S. 61–72.

82 Fechheimer 1914, S. 2. Hierzu grundlegend Peuckert 2014.

83 Schäfer 1919; ders. 1928.

84 Fechheimer 1914, S. 4.

85 Schäfer 1928, S. 18. Im hier zitierten Aufsatz bringt Schäfer selbst jedoch vor allem seine Skepsis gegenüber diesen Entlehnungen der modernen Kunst zum Ausdruck.

86 Die Frage, ob sich altägyptische Bildwerke überhaupt adäquat mit den in der europäischen Neuzeit entstandenen Konzepten *Kunst* und *Ästhetik* beschreiben und analysieren lassen, ist damit freilich nicht berührt. Zu dieser Diskussion zuletzt Widmaier 2017; anders u. a. Baines 2007; Verbovsek 2011.
